# 

**Published:** 1859-04

**Authors:** 


*Michigan State Dental Association.*—The fourth annual meeting of this association, as reported in the Dental Reporter, was held in Detroit on January 11th and 12th, 1859. The following subjects were discussed:—

1. Preparing cavities. 2. Filling teeth. 3. Treatment of exposed nerves. 4. Alveolar abscess. 5. Mechanical dentistry. 6. Getting up casts. 7. Artificial teeth.

---

*Indiana State Dental Convention.*—We have lately received a report of the meeting of this Convention, made for the New York Dental Journal and Reporter, but we are unable to gather any idea of its date—the only reference to a date being the one on second line of the report, which states that "in pursuance of a call made at a preliminary meeting held on the 18th September last, in the city of Indianapolis, a large number of the profession of that and other states, met in convention at Ramsey's Hall, for the purpose of forming a State Dental Association." Our readers will, therefore, as we have no other means of discovering the fact, excuse us if we are not able to give them further definite information as to the time when this society was founded.

Dr. J. F. Johnson, chairman of the preliminary meeting, made an address; a constitution was offered and adopted; the officers were elected, and after some further parliamentary business the convention discussed the following questions:

1. Best means of preserving the teeth. 2. Treatment of exposed nerves. 3. The best means to correct irregularities of the teeth. 4. Mechanical dentistry. 5. Filing teeth. 6. Miscellaneous questions.

We trust that the future deliberations of this convention may not be characterized by such remarks as the following:—one of the speakers "said he was a 'blab,' especially when a subject was presented that he had made a 'hobby,' as he had in the use of crystal foil for filling teeth. He said the first thing with him was to make his patients think he was 'some pumpkins' of a dentist, and he was vain enough to think there were those in New York that thought he 'was some.' . . . ." The repeated use of vulgarisms of the worst description, of this person, forbids the extenuation of misprinting. His vanity must be great, indeed, if he thinks that he rises in the estimation of patients by the use of such eloquence.

*Our Contributors.*—We are glad to inform our readers that we have secured, since the publication of the last number of the Journal, the regular co-operation of fifty able contributors, among whom are many eminent dental practitioners and medical men of the first distinction. This number does not, however, include numerous foreign correspondents, from whom we hope to receive constant communications. Our object hereafter, will be to present a much larger and more varied original department.

---

*To the Profession.*—Those gentlemen of the profession who have the opportunity, would advance science, and confer a particular favor by forwarding to Christopher Johnston, M. D., Professor of Miscroscopical and Comparative Anatomy of the Teeth, in the Baltimore College of Dental Surgery, any specimens of teeth of *recent* or *fossil animals*, or fossil bones, attaching to them the locality.

